# An external facilitation intervention to increase uptake of an adverse drug event reporting intervention

**DOI:** 10.3389/frhs.2023.1106586

**Published:** 2023-06-02

**Authors:** Erica Y. Lau, Serena S. Small, Kate Butcher, Amber Cragg, Gabriel W. Loh, Steve Shalansky, Corinne M. Hohl

**Affiliations:** ^1^ Department of Emergency Medicine, University of British Columbia, Vancouver, BC, Canada; ^2^ Vancouver Coastal Health Research Centre, Centre for Clinical Epidemiology and Evaluation, Vancouver, BC, Canada; ^3^ Pharmaceutical Science, Vancouver General Hospital, Vancouver Coastal Health, Vancouver, BC, Canada; ^4^ Richmond Hospital Pharmacy Department, Lower Mainland Pharmacy Services, Richmond, BC, Canada; ^5^ Pharmacy Department, St. Paul’s Hospital, Providence Health Care, Vancouver, BC, Canada

**Keywords:** contextual factors, core functions and forms, adverse drug events, facilitation, health information technology (Health IT), implementation strategies

## Abstract

**Background:**

Adverse drug events (ADEs) are a leading cause of emergency department visits and hospital admissions in Canada. ActionADE prevents repeat ADEs by enabling clinicians to document and communicate standardized ADE information across care settings. We used an external facilitation intervention to promote the uptake of ActionADE in four hospitals in British Columbia, Canada. This study examined whether, how and in what context external facilitation influenced the uptake of ActionADE.

**Methods:**

In this convergent-parallel mixed-methods study, an external facilitator used a four-step iterative process to support site champions using context-specific implementation strategies to increase the ADE reporting rate at their sites. We extracted archival data to assess implementation determinants before and after the implementation of the external facilitation and implementation strategies. We also retrieved data on the mean monthly counts of reported ADEs for each user from the ActionADE server. Zero-inflated Poisson models were used to examine changes in mean monthly counts of reported ADEs per user between pre-intervention (June 2021 to October 2021) and intervention (November 2021 to March 2022) periods.

**Results:**

The external facilitator and site champions co-created three functions: (1) educate pharmacists about what and how to report in ActionADE, (2) educate pharmacists about the impact of ActionADE on patient outcomes, and (3) provide social support for pharmacists to integrate ADE reporting into clinical workflows. Site champions used eight forms to address the three functions. Peer support and reporting competition were the two common strategies used by all sites. Sites’ responses to external facilitation varied. The rate of mean monthly counts of reported ADEs per user significantly increased during the intervention period compared to the pre-intervention period at LGH (RR: 3.74, 95% CI 2.78 to 5.01) and RH (RR: 1.43, 95% CI 1.23 to 1.94), but did not change at SPH (RR: 0.68, 95% CI: 0.43 to 1.09) and VGH (RR: 1.17, 95% CI 0.92 to 1.49). Leave of absence of the clinical pharmacist champion and failure to address all identified functions were implementation determinants that influenced the effectiveness of external facilitation.

**Conclusion:**

External facilitation effectively supported researchers and stakeholders to co-create context-specific implementation strategies. It increased ADE reporting at sites where clinical pharmacist champions were available, and where all functions were addressed.

## Background

Adverse drug events (ADEs)—harmful and unintended events related to medication use—are a leading cause of patient harm, and a burden on health systems (1–4). One in nine adult visits to the emergency department is caused by an ADE. Of those visits, one in three are repeat events (5). Repeat ADEs occur because clinicians may be unaware of patients' ADE histories when prescribing. Different health settings, such as hospitals, long-term care facilities and clinics, often use different clinical information systems that do not automatically exchange ADE information, leading to information discontinuity (5). Effective system-level interventions are needed to address this communication gap (6).

ActionADE is software that enables healthcare providers to document and share ADE information using standardized terminologies in a user-friendly electronic format (7–9). ActionADE (8) has been integrated with British Columbia's provincial medication dispensing database, PharmaNet, to automatically share ADE information documented in hospitals, where patients with severe and acute ADEs commonly seek care. This allows care providers in other health sectors (e.g., community clinics and pharmacies) across the province who have access to PharmaNet to access ADE information. Through systems integration, PharmaNet presents community pharmacists with standardized ADE alerts if they attempt to re-dispense a medication or medication of the same class for which the patient has an ADE recorded in PharmaNet. Preliminary data shows that ActionADE prevents repeat ADEs in 10.8% of patients with reports shared to PharmaNet (10), supporting the preliminary effectiveness of ActionADE in preventing re-exposure to culprit medication”.

Noteworthy, valuable clinical interventions scarcely implement themselves. The use of effective strategies to implement evidence-based interventions into clinical practice is necessary to ensure that patients receive the benefit (11). Implementation strategies are methods or techniques used to improve adoption, implementation, sustainment, and scale-up of interventions (12). The field of implementation science has made significant progress to generate evidence for implementation strategies in the past two decades, with published reviews and taxonomies describing over 70 strategies, such as audit and feedback and educational outreach (12–16). Selecting the most appropriate implementation strategies for clinical interventions requires thorough understanding of implementation determinants (i.e., barriers and enablers) across multiple levels of stakeholders and settings in the dynamic and complex healthcare system (15, 17, 18) However, the literature offers limited evidence on methods for doing so effectively (17, 19, 20).

External facilitation offers a promising approach to align implementation strategy with determinants. External facilitation is a multi-faceted process whereby external implementation experts work with stakeholders to promote interactive problem-solving and knowledge exchange that supports the adoption and use of an evidence-based practice (21–23). Key components of external facilitation include assessing the contexts, assisting teams in identifying problems and developing implementation strategies, monitoring, and providing feedback around the change efforts (24–26). External facilitation has been effective in improving the uptake of various health interventions such as antenatal care (27), postpartum care, peer specialist service (28), opioid use disorder treatment (29), and psychosocial intervention for homelessness (30). There is growing evidence suggesting that external facilitation is effective in improving health intervention implementation (29). However, most studies did not provide clear and explicit descriptions of the facilitation process, which prevented others from repeating and adapting this approach. A systematic review synthesized evidence from 195 facilitation studies to identify the role and characteristics of facilitation, and found only six studies explicitly described the actual process (21). Moreover, we know little regarding context-specific effectiveness, particularly within multi-site interventions (25, 31). Previous multi-site studies found that the effects of external facilitation on intervention uptake were variable across sites, but the factors contributing to such variations has yet to be identified (28, 29, 32).

The objectives of this study were to examine whether, how and in which context external facilitation influenced the uptake of ActionADE. We aimed to address four research questions:

1. What were the implementation determinants that influenced uptake of ActionADE before the external facilitation?
2. What implementation strategies were used by each site to promote ActionADE uptake?
3. What were the effects of external facilitation on the mean monthly counts of reported ADEs per user?
4. What were the implementation determinants that influenced uptake of ActionADE during external facilitation?

## Methods

### Study design

We used a convergent-parallel mixed-methods design. We collected quantitative data from archival data and qualitative data from meeting notes during the study period. We analyzed quantitative and qualitative results separately and then triangulated the findings when interpreting the results (33).

### Setting

Since December 2020, nine hospitals have adopted ActionADE, with four engaging in active change management to onboard new users and sustain reporting. After a 10-month pilot implementation (January to October 2021) to secure stakeholder buy-in, we initiated an external facilitation intervention to increase the uptake of ActionADE among frontline providers. We presented the characteristics of the four participating hospitals in Table 1. The four participating hospitals were Lions Gate Hospital (LGH), Richmond Hospital (RH), St Paul's Hospital (SPH) and Vancouver General Hospital (VGH). All are in the Greater Vancouver area within the Vancouver Coastal Health Authority. All are acute care hospitals serving urban areas, and each had two pharmacists coordinating the implementation of ActionADE. LGH and RH are smaller urban community hospitals with fewer emergency department visits, fewer clinical areas covered by pharmacists, and fewer onsite clinical pharmacists. SPH and VGH are tertiary and quaternary urban teaching hospitals, respectively. All sites were involved in developing ActionADE, with pilot testing occurring at VGH.

**Table 1:** Site characteristics.

Site	# of beds and types	Population served	# of emergency department visits/year	Clinical areas covered by pharmacists	# of pharmacists	# of registered ActionADE users	Implementation team composition
Lions Gate Hospital (LGH)	268 beds, acute care, community hospital	Urban and rural	65,000	10	27	27	1 clinical pharmacist and 1 clinical pharmacy coordinator
Richmond Hospital (RH)	200 beds, acute care, teaching hospital	Urban	50,000	8	29	22	1 clinical pharmacist and 1 clinical pharmacy coordinator
St. Paul's Hospital (SPH)	548 beds, acute care, teaching hospital	Urban	123,000	25	69	69	1 clinical pharmacist and 1 clinical pharmacy coordinator
Vancouver General Hospital (VGH)	1,900 beds, acute care, teaching hospital	Urban	94,348	25	64	64	1 clinical pharmacist and 1 clinical pharmacy coordinator

### External facilitation intervention

We conducted external facilitation between November 2021 and March 2022. External facilitation aimed to increase the use of ActionADE by supporting site champions to develop and use implementation strategies that fit their contexts. We hypothesized that the external facilitation process would lead to the use of context-specific strategies and increase mean monthly counts of reported ADEs per user.

**Figure 1:**
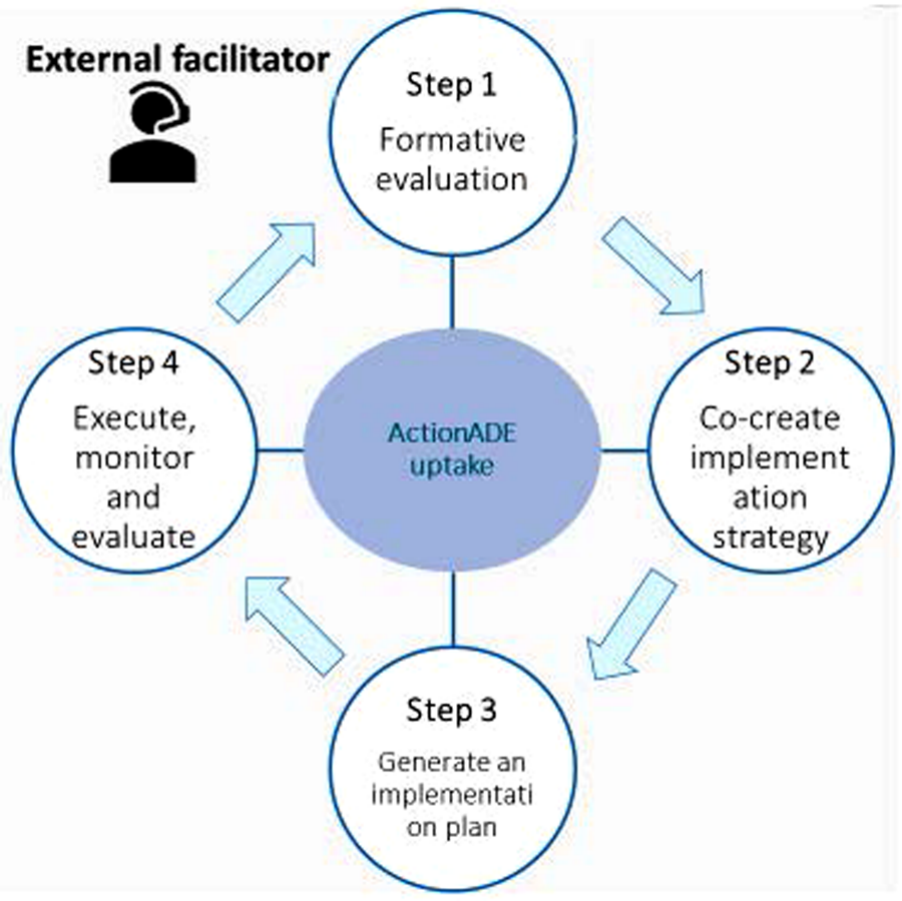
The four iterative steps of the external facilitation intervention for promoting uptake of ActionADE.

A research team member (EL) with training in implementation science and knowledge about the implementation settings served as the external facilitator (EF) to provide strategic and methodological support to site champions. EF used a four-step iterative process guided by previous facilitation studies (Figure 1) (29, 34).

#### Step 1: formative evaluation

The formative evaluation aimed to identify implementation determinants (i.e., factors that influence implementation success or failure) (35) influencing pharmacists' use of ActionADE before the external facilitation intervention. To identify implementation determinants, the EF analyzed meeting minutes during the implementation planning and pilot implementation phases. The EF then categorized the identified implementation determinants according to the Consolidated Framework for Implementation Research (CFIR) (36). Next, the EF met with champions at each site to refine the list of identified implementation determinants.

#### Step 2: co-create implementation strategies

To develop implementation strategies, the EF met with site champions to co-create a list of strategies targeting determinants identified in step 1. They then operationalized the strategies by specifying the name (10), purposes, action (the specific activities or processes that need to be enacted), the actors (who acts the strategy), and action target (target population of the actions) (37).

#### Step 3: generate an implementation plan

The EF developed, discussed, and refined an implementation plan with all site champions, which outlined the context, purpose, scope, timeline, target outcomes, and implementation strategies to increase the uptake of ActionADE. The plan was a living document for the EF to provide updates on ActionADE usage and document changes in implementation strategies and contextual factors that influence implementation at each site. The EF shared the plan with the site champions electronically.

#### Step 4: execute, monitor, and evaluate

Site champions executed the implementation plan, while the EF and research team monitored the process and evaluated outcomes through bi-weekly emails and monthly meetings. The EF met with site champions monthly to review utilization statistics, revisit implementation determinants, and modify the implementation strategies, if needed. During the post-intervention period, the EF met with champions at each site to obtain feedback for the external facilitation intervention and discuss determinants identified at pre-intervention or that emerged during the intervention.

### Outcome measures

Qualitative outcomes were implementation determinants reported before and after implementation of the external facilitation intervention, the functions and forms of the implementation strategies, and contextual factors influencing the uptake of ActionADE during the intervention. We extracted data on the implementation determinants before the intervention from meeting minutes documented between December 2020 to October 2021 (ActionADE pilot implementation period). The research team used a template for recording meeting minutes. A research team member recorded the date, time, purposes, and attendees of the meeting. The note-taker also recorded key discussion points, decisions, and action items. We extracted data on the implementation strategies used at each site and contextual factors from meeting notes documented between November 2021 and May 2022 (during the external facilitation intervention). These meeting notes captured opinions from research team members, patient partners, pharmacists at the participating hospitals, and site champions. Monthly meetings embedded within the external facilitation intervention offered a conducive environment for site champions to recall their implementation strategies. This approach was ideal for obtaining frequent feedback and specific perspectives on time-sensitive issues.

The main quantitative outcome was the mean monthly count of reported ADEs per user. We retrieved data on the mean monthly counts of reported ADEs for each individual user from the ActionADE server between June 2021 and March 2022 (10 months). We included data from pharmacists who registered for an ActionADE account before 1 June 2021 and held active employment without leaves at the same hospital throughout the study period.

### Data analysis

We analyzed qualitative data by thematically summarizing the meeting minutes. We coded the implementation determinants and the contextual factors according to the Consolidation Framework for Implementation Research (CFIR) qualitative data codebook (38). To describe implementation strategies, we drew upon the concepts of functions and forms, a crucial concept to guide the development of complex, adaptable and scalable innovations (39, 40). Functions are the purpose of a set of activities, why it matters and how it produces changes in the expected outcomes. Forms are a set of activities used to meet the functions (37, 39, 41). For example, in the context of ActionADE, a function could be educating pharmacists on what to report in ActionADE. The form for the first site could be delivering information about ActionADE reporting criteria to pharmacists in a group presentation, while the form for the second site could be delivering the same information through a user manual. The EF shared a summary of the qualitative findings with research team members, and the team subsequently reached a consensus about the implementation strategies and determinants through discussion. Qualitative analyses were conducted using NVivo 11 qualitative data analysis software (QSR International).

For quantitative data, we used descriptive statistics to calculate the means and standard deviations. We measured the effects of the external facilitation on the mean monthly counts of reported ADEs per user using zero-inflated Poisson models. We selected this model because exploratory analyses showed that the distribution of participants' mean monthly counts of reported ADEs was overdispersed (i.e., mean and variance differ significantly) and contained an excess of zeros created by non-adopters (42). To account for these issues, the model optimizes the estimations by creating two regression equations: the logit component for predicting excess zero counts and the typical Poisson component for predicting differences in the occurrence of the count (42, 43).

Given the heterogeneity of site characteristics and implementation strategies, we stratified the analysis by site. The model included the mean monthly counts of reported ADEs per user between June 2021 to March 2022 as the dependent variable and time as the independent variable. We treated time as a categorical variable, with 0 indicating the pre-intervention period (June 2021 to October 2021) and 1 for the intervention period (November 2021 to March 2022). We also tested a random effect term to account for repeated measurements nested within users. The random effects were not statistically significant in models for LGH, SPH and VGH. The model did not converge for RH's model likely due to a small sample size. Therefore, we removed the random effect term in the final models for RH. We validated the model by plotting the predicted and observed residual values from the models. The level of significance was set at *p* < 0.05. We conducted quantitative statistical analyses using SAS 9.4 (SAS).

The model produced two sets of estimates: a logistic component that yielded the odds ratios predicting the odds of having zero monthly counts of reported ADEs per user, a Poisson component that yielded the rate ratios (RRs) of the mean monthly counts of reported ADEs per user between the pre and during the intervention period after adjusting for excess zeroes by the logistic component (43). With a focus on the effects of the external facilitation on the mean monthly counts of reported ADEs per user, hereinafter, we presented and interpreted the RRs from the Poisson component only.

## Results

### Research question 1: what were the implementation determinants that influenced uptake of ActionADE before the external facilitation?

The formative evaluation identified four categories of implementation determinants that were common across sites: available resources, compatibility with workflow, relative priority and providers' knowledge and belief.

#### Available resources

All site champions noted lack of dedicated staff time as a major barrier to implementing ActionADE. They noted that staff shortages and turnover impacted reporting. Site champions at RH and LGH stated that they were smaller hospitals with fewer resources per patient compared to other sites.

#### Compatibility

Site champions stated that pharmacists had difficulties fitting ActionADE into their existing workflows. At the time of the study, pharmacists were unable to directly access ActionADE in the health information system being used without searching for it or receive visual reminders for ADE reporting through their local electronic medical records systems. Without streamlining the process, site champions felt that pharmacists were uncertain about the stage during care provision they should integrate ADE reporting into their workflow. When a patient transitioned between care areas (e.g., from the emergency department to an in-patient ward) there was no mechanism to support the handover of patients' ADE information across service locations.

#### Relative priority

At the time of the intervention new initiatives, such as COVID-19 vaccinations and training of new hires (due to the high staff turnover rate), competed with ActionADE implementation activities. With staff shortages, pharmacists were stressed, and experienced burnout and change fatigue. In this context, the site champions noted that pharmacists might have been less likely to prioritize ADE reporting.

#### Providers' knowledge and belief

Site champions noted that some pharmacists had questions about the types of ADEs to report (e.g., non-adherence, refuted allergy) and about specific data fields. Site champions noted that some pharmacists had not yet seen the impact of ADE reporting on patient care. Site champions suggested that these perceptions may explain why some pharmacists were reluctant to adopt the intervention.

### Research question 2: what implementation strategies were used by each site to promote ActionADE uptake?

During step 2 of the external facilitation process, the EF and site champions co-created functions and forms for the implementation strategies based on the implementation determinants identified in step 1. The EF and site champions recognized the complexity of addressing implementation determinants related to available resources, relative priority and compatibility of ActionADE with other health information systems. Increasing the number of pharmacists and changing organizational priorities for ADE reporting were not feasible functions, and beyond the capacity of the research team. Similarly, more fulsome integration into other health information systems requires infrastructure from multisectoral collaboration (e.g., data standards, data privacy regulations and technological infrastructure), which could not be accomplished over the course of five months. Due to these constraints, site champions suggested improving pharmacists' education around the clinical impact that ActionADE could have on patient outcomes to motivate them to prioritize time for ADE reporting and providing social support for pharmacists to integrate ActionADE into clinical workflow. Table 2 describes the three functions co-created by the EF and site champions: (1) educate pharmacists about what and how to report in ActionADE, (2) educate pharmacists about the impact of ActionADE on patient outcomes and (3) provide social support for pharmacists to integrate ActionADE into clinical workflow. We operationalized social support as supports accessible to an individual through social ties to other individuals and groups, such as encouragement from a co-worker (44).

**Table 2:** Implementation strategies (functions and forms) used by each site during the external facilitation.

Determinant	Function	Form (Name of the strategy)	Form (Actor Actions and Action Target*)
Providers’ knowledge about ActionADE (uncertain about what and how to use ActionADE)	1. Educate pharmacists about what and how to report in ActionADE	1.1 Conduct educational meetings	***LGH, RH, SPH****:* Research team delivered a 1-hour presentations covering why, what, and how to report in ActionADE, recent ADE examples, most reported drug types documented in ActionADE and a quick demonstration.
			***VGH****:* not used.
		1.2 Develop and distribute educational materials	***LGH****:* Research team developed a new 1-page how-to guide. The champions distributed the materials on intranet
			***RH****:* Not used
			***SPH****:* Research team developed a new PowerPoint slide deck on how to access and use ActionADE. The champion presented it in a pharmacist meeting.
			***VGH****:* Champions re-distributed lanyard cards (include access information) and previously developed ActionADE materials (i.e., user guide, demonstration videos Q&A fact sheets) *via* intranet.
Providers’ belief, available resources, and relative priority	2. Educate pharmacists about the impact of ActionADE on patient outcomes.	2.1 Involve patients	***LGH, RH, SPH****:* A 1-hour presentation Patient partners shared ADEs experiences of family members and their perspectives on the importance of ActionADE on improving patient safety and quality of care.
			***VGH****:* not used
Compatibility	3. Provide social support for pharmacists to integrate ADE reporting into clinical workflow	3.1 Peer support	***LGH****:* Champions encouraged, reminded, and assisted individual pharmacists to use ActionADE in the emergency department and during weekly meetings.
			***RH****:* Champions to encourage, remind, and assist individual pharmacists to use ActionADE in different service areas and promoted ActionADE in weekly meetings.
			***SPH****:* Champions encouraged, reminded, and assisted pharmacists to use ActionADE in regular pharmacist meetings.
			***VGH****:* Champions met with individual pharmacists to encourage, remind, and assist them to use ActionADE.
		3.2 Identify and prepare additional champions	***LGH, SPH, VGH****:* Not used.
			***RH****:* Champions trained casual pharmacists to support ADE reporting.
		3.3 Visual cues	***LGH, SPH****:* Not used.
			***RH****:* Champions developed a poster associating ADE reporting with a routine practice --allergy reporting. The poster was displayed it in the pharmacy, on-call room, and medical room.
			***VGH****:* Champions wore an ActionADE button on scrub, displayed ADE pharmacist's contact information on emergency department phones, and a ActionADE posters in the pharmacy.

* The action targets were individual clinical pharmacists unless specified otherwise. LGH, Lion's Gate Hospital; RH, Richmond Hospital; SPH, St. Paul's Hospital; VGH, Vancouver General Hospital.

Site champions developed and used eight distinct forms to address the three functions (Table 2). Noteworthy, LGH, RH and SPH delivered forms meeting all three functions, while VGH addressed functions 1 and 3 only. All sites employed two common forms: peer support and reporting competitions. Peer support included site champions providing reminders, verbal encouragement and troubleshooting to pharmacists at their sites. Reporting competitions consisted of one individual-based and two team-based challenges in which pharmacists competed for prizes awarded to the top three reporters across sites individually or with a team of 2 to 3 members from the same site. Winners of the reporting competitions received gift cards to redeem for merchandise. Both forms encouraged pharmacists to integrate ActionADE into their clinical workflow by creating a social milieu for ADE reporting. Each site used slightly different forms to address the functions to fit their contexts. For instance, LGH employed educational meetings and materials to address function 1, while VGH used educational materials and 1-on-1 follow-up.

Each site operationalized the same form slightly differently. All sites used peer support but targeted different sub-groups. LGH focused on pharmacists in the emergency department; RH targeted pharmacists at different service locations; SPH targeted all clinical pharmacists, and VGH focused on less frequent users. Pharmacists were the action targets for forms. Site champions were the primary actors for most forms, with the research team assisting in the deployment. For instance, three site champions identified the need to develop and re-distribute ActionADE educational materials. The site champions were the ones who decided the content, format, and distribution channels of the educational materials. The research team supported them by sharing existing educational materials or tailoring new materials as requested.

### Research question 3: what were the effects of external facilitation on the mean monthly counts of reported ADEs per user?

**Figure 2:**
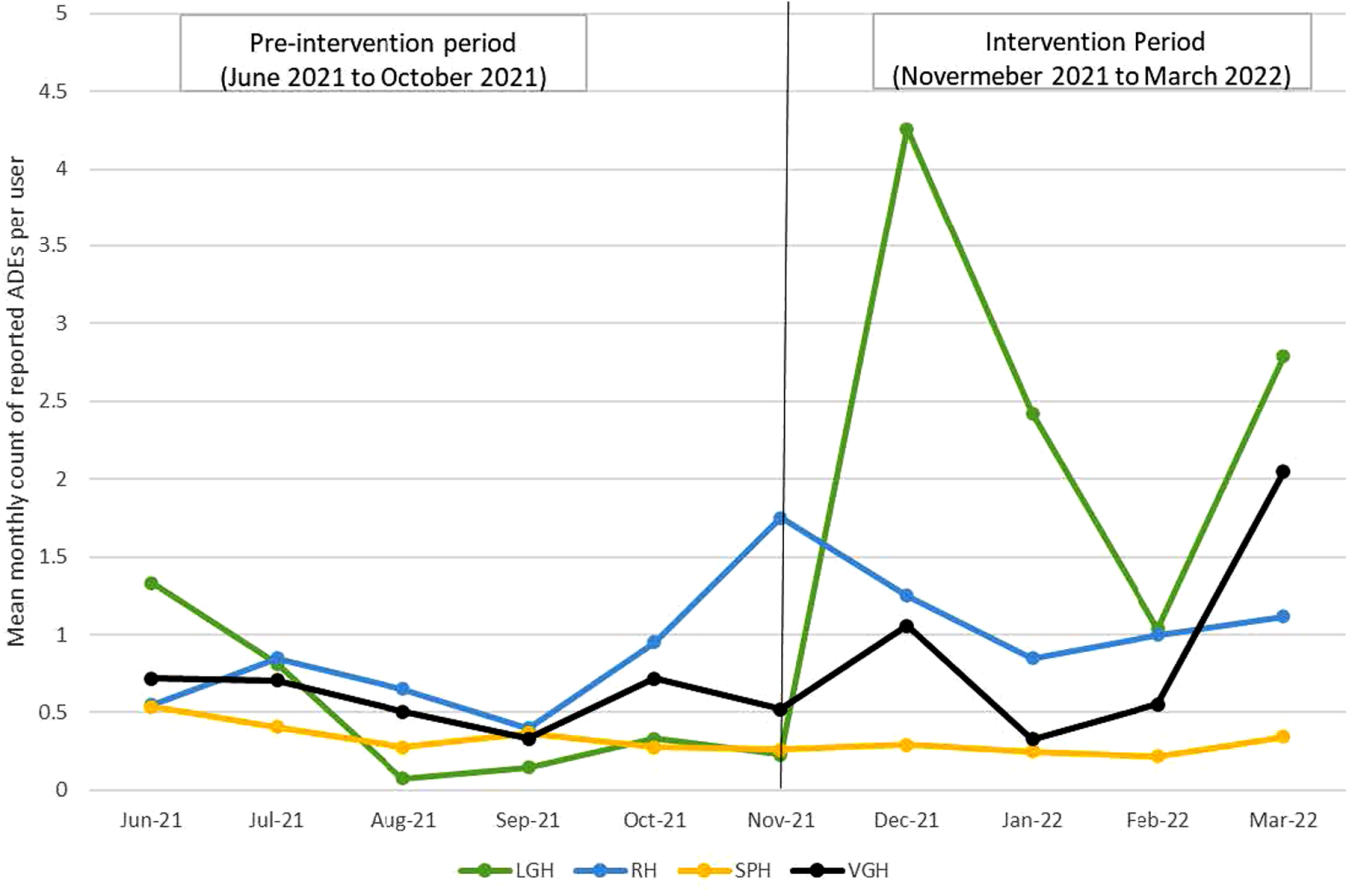
Mean monthly counts of reported ADEs per user at pre-intervention and intervention period by site.

The analytical sample included 146 pharmacist users and 1,460 observations. The mean monthly counts of reported ADEs per user were 0.57 ± 1.24 at pre-intervention compared to 0.94 ± 3.23 during the intervention period. The mean monthly counts of reported ADEs per user were steady during the pre-intervention period and fluctuated during the intervention period across all sites; the counts for LGH, RH and VGH reached the peak during the intervention period (Figure 2).

Results from modelling showed that the rate of mean monthly counts of reported ADEs per user were significantly higher during the intervention period at LGH and RH, but null results were observed at SPH and VGH. The rate of mean monthly counts of reported ADEs per user during the intervention period was 3.74 times (RR: 3.74, 95% CI 2.78 to 5.01), 1.43 times (RR: 1.43, 95% CI 1.23 to 1.94) the rate for the pre-intervention period at LGH and RH, respectively. There was no difference in the rate between the pre-intervention and intervention periods at SPH (RR: 0.68, 95% CI: 0.43 to 1.09) and at VGH (RR: 1.17, 95% CI 0.92 to 1.49) (Table 3). Model validation plots showed that the observed and predicted values aligned closely, indicating that models fit the data well (data not shown).

**Table 3:** Rate ratios of mean monthly counts of reported ADEs during the pre-intervention vs. intervention periods for the total sample and by site.

Variable	LGH	RH	SPH	VGH
	Rate Ratio (95% CI)	Rate Ratio (95% CI)	Rate Ratio (95% CI)	Rate Ratio (95% CI)
**Time**				
Pre-intervention period (Jun 2021—Oct 2021)	*Reference*	*Reference*	*Reference*	*Reference*
Intervention period (Nov 2021—Mar 2022)	3.74 (2.78 to 5.01)	1.43 (1.23 to 1.94)	0.68 (0.43 to 1.09)	1.17 (0.92 to 1.49)

### Research question 4: what were the implementation determinants that influenced uptake of ActionADE during the external facilitation?

During and after the external facilitation, the EF discussed implementation determinants that may have influenced the ADE reporting rates and corresponding solutions.

#### Available resources

As with the pre-intervention period, lack of dedicated staff time was the most frequently discussed barrier. Due to a higher rate of staff turnover and sick calls during the COVID-19 pandemic, clinical pharmacists faced higher workloads. This issue impacted not only ActionADE use but other patient care activities more broadly. The SPH site champion noted that staff turnover issues had impacted the reporting rates significantly during the intervention period because one clinical pharmacist champion went on parental leave unexpectedly early right after the external facilitation intervention began. The other site champion had limited time to move implementation activities forward.

#### Providers' knowledge and belief

Our qualitative data showed that discussions around providers' knowledge about ActionADE were less frequent after implementation of the external facilitation intervention. Site champions had noted that only a few pharmacists had questions about the eligibility of reporting for specific cases and duplicate reports. Regarding providers' beliefs, site champions reported that some pharmacists hesitated to use ActionADE because they feared that their reports would “scare prescribers” and take away necessary medications that should be re-dispensed.

#### Relative priority

Pharmacists were pulled into different initiatives during the intervention period, which may have influenced pharmacists' willingness and ability to use ActionADE. For example, the champion at LGH mentioned that pharmacists tended to prioritize treatment over preventive work. The champions at SPH also noted that the hospital prioritized admitted patients and hired a team of pharmacists to review their medications. However, many ADEs were identified in patients who were discharged from the ED who were not prioritized for medication review by some sites. Site champions also mentioned low levels of physician engagement may have prevented pharmacists from prioritizing ADE reporting in a team-based approach.

Other determinants included discontinuation of reporting competitions that had been designed to create a social milieu to stimulate ADE reporting. Site champions also suggested that regular reporters' work rotation schedules led to fluctuating monthly ADE report counts over time. While pharmacists were on a rotation with dispensary shifts, they rarely saw patients and would not encounter ADEs.

## Discussions

This mixed-methods study examined whether, how, and in which contexts external facilitation increased the uptake of ActionADE. Consistent with previous research, we found that external facilitation was effective in increasing the uptake of ActionADE, but effects varied by sites (28, 29, 32). We observed significant increase in ADE reporting at LGH and RH but null effects at SPH and VGH. The significant intervention effects at LGH and RH suggested external facilitation can be effective in improving intervention uptake by assisting clinical teams in developing tailored strategies based on the implementation determinants. The EF and site champions co-created three functions during the external facilitation process. They included educating pharmacists about what and how to report in ActionADE, educating pharmacists about the impact of ActionADE on patient outcomes and providing social support for pharmacists to integrate ADE reporting in the clinical workflow. The identified functions were similar to the recommended practices for implementing new digital services into the routine work of healthcare professionals by Nadva et al. (45). We added value to the existing literature by providing a menu of forms for each function, which future studies can adopt, test and adapt. Developing functions and the corresponding menu of forms is important for others to replicate an intervention or implementation strategy. Very few studies have provided explicit guidance on adapting an evidence-based practice to fit local contexts (46). Specifying the functions and forms of an intervention or implementation strategy provides other researchers or practitioners with explicit guidance and options about which adaptations to the intervention's form are allowable while preserving fidelity (46, 47).

The positive intervention effect was more profound in LGH than in RH. We did not observe differences in implementation strategies or determinants between the two sites. Thus, we attributed the variable effects to other factors not measured in this study. A potential factor could be the characteristics of individual users. Compared to RH, users in LGH appeared to be more responsive to the strategies, particularly during the months with patient partner presentations and reporting competitions. This speculation is consistent with previous research. Rycroft-Malone et al. (48) found that individual characteristics are prominent in the interaction between context and strategies. Staff members' learning skills and motivation significantly influenced the effectiveness of facilitation on research uptake. We attempted to survey users' perceptions of the implementation strategies, but the response rate was very low amid the pandemic. Future studies are needed to explore how user characteristics interact with determinants at different levels (e.g., organizations level) in influencing the process and effectiveness of external facilitation.

The null effects in the other two sites provided insights into the contexts in which external facilitation was less effective. We attributed the null intervention effect at VGH to the failure to address all the identified functions. VGH was the only site that did not address function 2, which was to educate pharmacists about the potential impact of ActionADE on patient outcomes. The null intervention effects at VGH suggested that all three functions identified through the external facilitation must be addressed to achieve the expected outcome. Gustavson and colleagues (29) examined the effects of external facilitation on increasing use of medication treatment for opioid use disorder in nine veteran health administration facilities. They observed a significant increase in program uptake in facilities who achieved almost all the implementation goals. Previous evidence also supported that perceived benefits of the intervention were an important determinant for changing clinical practices among healthcare professionals (36, 49, 50). Future studies with a larger sample size and experimental design are needed to verify this finding.

SPH used a similar set of forms as LGH and RH but did not result in a significant improvement in ADE reporting rate. Our qualitative data suggested that the leave of absence of a key site champion during the external facilitation intervention may have attributed to the null intervention effects. As mentioned by the SPH champion, the absence of the clinical pharmacist champion substantially limited the execution of implementation activities and engagement with other pharmacists. This finding was not surprising because previous research consistently indicated that use of program champions was a critical implementation determinant for healthcare interventions (51–53). Two randomized trials (54, 55) tested the impact of program champions on changing clinical practices in healthcare professionals. McCabe et al. (54) found that the presence of a formally identified, designated champions was associated with an increase in residential aged care staff sensitivity to depression among residents. Bentz et al. (55) reported that the presence of clinical champions associated with an increased rate of referral to a state-level smoking quit line.

One interesting finding was that the external facilitation could not address several essential implementation determinants (i.e., staffing shortages, competing demands) that were out of our team's control, but it nonetheless achieved a significant improvement in ADE reporting at two sites. A plausible explanation was that being able to address other determinants, including providers' knowledge and belief, may have partially offset the negative impact from staffing shortages and competing demands. Previous studies suggested that implementation determinants interact synergistically to influence implementation success. A determinant that is perceived as less influential may be a preceding factor to improve another determinant (56, 57). For example, in ActionADE, improving pharmacists' belief might be a preceding factor to address staff shortage issue. Once pharmacists recognized the impact of ActionADE in improving patient outcomes, they may have been more motivated to prioritize their time for ADE reporting. However, we need future studies to verify these speculations. In the current study, we were unable to fully integrate ActionADE into the electronic medical record workflow due to resource constraints. Future work should assess the effectiveness of improved workflow integration compared to other strategies.

## Reflections and lessons learned

When implementing ActionADE at multiple sites, adaptation to the local context was necessary to meet the diverse individuals' needs in order to avoid diminished intervention benefits (58). We found that external facilitation was an effective strategy to help implementation teams to identify the needs and focus of the adaptation. The functions and forms concept provided a new way of thinking when designing implementation strategies for a complex intervention undertaken in a complex health system. The function and form concept helped the team emphasize the intended function or purpose of the strategy instead of the dose (e.g., one or three training sessions). It also offered a practical tool for distinguishing between standardized and adaptable elements of the intervention or implementation strategies. However, conducting external facilitation was not without challenges. The external facilitation process was intense. It involved frequent communications with program champions, detailed records, and a rapid and timely evaluation-feedback loop. Nonetheless, the frequent contacts and in-depth evaluation of the contexts were beneficial for both parties to build a trusting relationship and co-create strategies that fit. The intense process was also necessary to keep the project on our site champions' agenda against other competing priorities. With the positive experiences, we decided to extend the facilitation intervention and continue to adapt and monitor changes in implementation strategies.

Our findings should be interpreted with the following limitations in mind. We conducted this study in four hospitals in one geographic location, limiting our findings' generalisability. Second, our qualitative data primarily captured the perspectives of the site champions, which may not be representative of all ActionADE users. We measured most of the implementation outcomes based on archival data. While this approach may not be able to explore a comprehensive list of implementation determinants, it provided a conducive and practical approach to capture longitudinal changes in providers' perceptions, contextual factors and implementation strategies. In this exploratory study, we did not assess fidelity of the implementation strategies. We need further studies to verify the effectiveness of the identified forms in addressing the functions and corresponding determinants.

## Conclusion

This study offers new insights on whether, how, and in what contexts external facilitation promoted the uptake of clinical intervention. Our findings showed that external facilitation can be effective in promoting the uptake of ActionADE across multiple hospitals by assisting clinical teams in developing tailored strategies (functions and forms) based on pre-assessed implementation determinants. However, its effectiveness varied depending on the site's ability to deliver the identified strategies and the emergence of new determinants. Future studies are needed to examine the long-term success of external facilitation and strengthen the evidence base regarding factors influencing effectiveness of external facilitation.

## Data Availability

The raw data supporting the conclusions of this article will be made available by the authors, without undue reservation.
